# Phospho-aspirin (MDC-22) inhibits breast cancer in preclinical animal models: an effect mediated by EGFR inhibition, p53 acetylation and oxidative stress

**DOI:** 10.1186/1471-2407-14-141

**Published:** 2014-02-28

**Authors:** Liqun Huang, Chi C Wong, Gerardo G Mackenzie, Yu Sun, Ka Wing Cheng, Kvetoslava Vrankova, Ninche Alston, Nengtai Ouyang, Basil Rigas

**Affiliations:** 1 Division of Cancer Prevention, Department of Medicine, Stony Brook University, Stony Brook, New York 11794-8173, USA; 2 Medicon Pharmaceuticals, Inc, Setauket, NY 11733, USA

**Keywords:** Breast cancer, Triple-negative breast cancer, Phospho-aspirin, Non-steroidal anti-inflammatory drugs, Epidermal growth factor receptor (EGFR), p53, Oxidative stress

## Abstract

**Background:**

The anticancer properties of aspirin are restricted by its gastrointestinal toxicity and its limited efficacy. Therefore, we synthesized phospho-aspirin (PA-2; MDC-22), a novel derivative of aspirin, and evaluated its chemotherapeutic and chemopreventive efficacy in preclinical models of triple negative breast cancer (TNBC).

**Methods:**

Efficacy of PA-2 was evaluated in human breast cancer cells in vitro, and in orthotopic and subcutaneous TNBC xenografts in nude mice. Mechanistic studies were also carried out to elucidate the mechanism of action of PA-2.

**Results:**

PA-2 inhibited the growth of TNBC cells in vitro more potently than aspirin. Treatment of established subcutaneous TNBC xenografts (MDA-MB-231 and BT-20) with PA-2 induced a strong growth inhibitory effect, resulting in tumor stasis (79% and 90% inhibition, respectively). PA-2, but not aspirin, significantly prevented the development of orthotopic MDA-MB-231 xenografts (62% inhibition). Mechanistically, PA-2: 1) inhibited the activation of epidermal growth factor receptor (EGFR) and suppressed its downstream signaling cascades, including PI3K/AKT/mTOR and STAT3; 2) induced acetylation of p53 at multiple lysine residues and enhanced its DNA binding activity, leading to cell cycle arrest; and 3) induced oxidative stress by suppressing the thioredoxin system, consequently inhibiting the activation of the redox sensitive transcription factor NF-κB. These molecular alterations were observed in vitro and in vivo, demonstrating their relevance to the anticancer effect of PA-2.

**Conclusions:**

Our findings demonstrate that PA-2 possesses potent chemotherapeutic efficacy against TNBC, and is also effective in its chemoprevention, warranting further evaluation as an anticancer agent.

## Background

Breast cancer is the second most common cause of female cancer-related deaths, with more than one million new cases diagnosed per year throughout the world [1]. Despite advances in its early detection, breast cancer remains a significant health problem. In particular, triple negative breast cancer (TNBC) is known to be more aggressive with poor prognosis, and is frequently associated with resistance to chemotherapeutic agents. Thus, novel agents capable of inhibiting TNBC are urgently needed.

**Figure 1:**
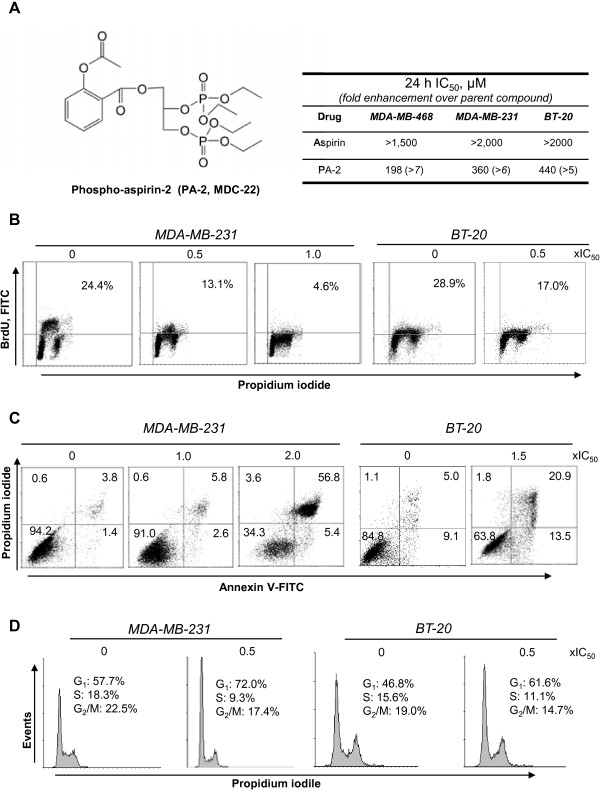
**Phospho-aspirin-2 inhibits the growth of TNBC cells. A: ***Left*: Chemical structure of phospho-aspirin-2 (PA-2, MDC-22). *Right*: 24 h-IC_50_ values of PA-2 and aspirin in TNBC cell lines. **B:** MDA-MB-231 and BT-20 cells were treated with PA-2 for 24 h and the percentage of proliferating cells was determined by BrdU incorporation. **C:** MDA-MB-231 and BT-20 cells treated with PA-2 for 24 h were stained with Annexin V/PI, and the percentage of apoptotic cells was determined by flow cytometry. **D:** PA-2 blocks the G_1_/S cell cycle phase transition after 24 h treatment in MDA-MB-231 cells, determined by flow cytometry following PI staining.

Aspirin, a prototypical non-steroidal anti-inflammatory drug (NSAID), is the most widely used anti-inflammatory medication in the world [2,3]. NSAIDs have a significant antineoplastic effect, which should be viewed, at least in part, in the context of the increasingly appreciated role of inflammation in cancer. Aspirin has been formally documented to be a chemopreventive agent against colon cancer [4,5]. Epidemiological studies also support a role of aspirin in reducing the risk of breast cancer [6]. However, gastrointestinal toxicity caused by chronic aspirin use is a significant health concern. In order to reduce the toxicity and enhance the efficacy of aspirin, we synthesized phospho-aspirin (PA-2; MDC-22; Figure 1A), which consists of aspirin chemically modified at its –COOH group, the moiety accounting for its gastrointestinal toxicity [7,8]. Indeed, as we have recently reported, the gastrointestinal toxicity of PA-2 in rats is much reduced compared to that of aspirin [9].

The epidermal growth factor receptor (EGFR) and p53 are key molecular determinants of TNBC [10-12]. Aberrant activation of EGFR plays an important role in breast carcinogenesis via the sustained initiation of downstream cascades that promote cell survival and proliferation. Thus, EGFR is an attractive target for the development of cancer therapeutics [13]. On the other hand, the inactivation of p53, a potent tumor suppressor, is also a major contributor to breast cancer development [12]. Apart from its ability to block cell cycle progression and promote apoptosis, it is now appreciated that p53 also suppresses tumor development by modulating autophagy, cellular metabolism, angiogenesis, and metastasis [14]. This portends that the restoration of p53 function in tumors will be extremely beneficial, since it will not merely inhibit the growth of tumor cells but also obliterate the microenvironment required for tumor survival.

Herein, we examined the antineoplastic properties of PA-2 in TNBC in vitro and in vivo. PA-2 was much more potent than aspirin in inhibiting the growth of TNBC cells and strongly suppressed TNBC growth in subcutaneous and orthotopic xenograft models. Mechanistically, the antineoplastic effect of PA-2 is mediated through inhibition of EGFR, acetylation of p53 and induction of oxidative stress.

## Methods

### Reagents

PA-2 was provided by Medicon Pharmaceuticals, Inc., Setauket, NY. Aspirin were purchased from Sigma (St Louis, MO). For cell culture study, we prepared 500 mM stock solutions of both in DMSO. In all cell culture media, the final DMSO concentration was adjusted to 1%. All general solvents and reagents were of HPLC grade or of the highest grade commercially available. Antibodies against β-actin were from Sigma. All other antibodies were from Cell Signaling (Beverly, MA).

### Cell culture

We used three human breast cancer cell lines: MDA-MB 231 (*ER-, PR-, HER2/Neu-, EGFR+, and p53 mutant R280K*), BT-20 ( *ER-, PR-, HER2/Neu-, EGFR++, and p53 mutant K132G*), and MDA-MB-468 (*ER-, PR-, HER2/Neu-, EGFR++, and p53 mutant R2073H*). All were obtained from the American Type Culture Collection (ATCC, Manassas, VA, and grown as monolayers in the specific medium and conditions suggested by ATCC. All cell lines were grown in our laboratory less than 6 months after their receipt and the cells studied were between passages 2-10.

### Cell viability assay

We used an assay based on reduction of 3-(4,5-dimethylthiazol-2-yl)-2,5-diphenyltetrazolium bromide dye (MTT), which was determined according to the manufacturer’s protocol (Promega, Madison, WI).

### Cytokinetic analysis

For apoptosis, cells were seeded and treated with PA-2 for 24 h, trypsinized and stained with Annexin V-FITC (100X dilution; Invitrogen, Carlsbad, CA) and PI (0.5 μg/ml; Sigma, St Louis, MO), then analyzed by FACScaliber (BD Biosciences, San Jose, CA). To determine cell proliferation, we measured the incorporation of 5-bromo-2′-deoxyuridine (BrdU) into newly synthesized cellular DNA followed by the manufacture’s protocol (BD Biosciences), and cells were subjected to flow cytometric analysis. Cell cycle phase distribution was analyzed by flow cytometry as described [15].

### Plasmid and siRNA transfection

EGFR, and SIRT1 plasmids were purchased from Addgene (Cambridge, MA). Transient transfection was performed with Lipofectamine 2000 (Invitrogen, Carlsbad, CA) following the manufacturer’s instructions.

### Determination of reactive oxygen and nitrogen species (RONS)

After the indicated treatment, cells were collected by trypsinization, resuspended in 10 μM of 5-(and-6)-carboxy-2′,7′-dichlorodihydrofluorescein diacetate (H_2_DCFDA Invitrogen), or MitoSox Red (Invitrogen) or dihydroethidium (DHE, Sigma), incubated at 37°C for 30 min in the dark and their fluorescence intensity was determined by flow cytometry.

### Urinary F_2_ isoprostane assay

Urine was collected at the endpoint of treatment. Levels of F_2_ isoprostane and creatinine in urine were determined by ELISA (Oxford Biomedical Research, MA). F2-isoprostane values were normalized to creatinine levels.

### Determination of TrxR reductase activity

After treatment, cells were lysed and TrxR activity was determined in the protein lysate using a commercially available kit, following the instructions of the manufacturer (Cayman Chemical, Ann Arbor, MI). In this assay, TrxR uses NADPH to reduce 5,5′-dithiobis-(2-nitrobenzoic acid) to 5-thio-2-nitrobenzoic acid (TNB). Glutathione (oxidized and reduced) was determined by the glutathione (GSH) reductase-coupled 5,5′-dithiobis(2-nitrobenzoic acid) assay [16].

### Immunoblotting

After treatment with PA-2 as indicated, cells were scraped on ice, washed with ice-cold PBS and lysed in RIPA lysis buffer (Sigma). Protein concentration was determined using the Bradford method (Bio-Rad, Hercules, CA). Electrophoresis of cell lysates were performed on 10% SDS-polyacrylamide gel electrophoresis gels and protein was transferred onto nitrocellulose membranes as described [17];

### Electrophoretic Mobility Shift Assay (EMSA)

Following treatment, nuclear fractions were isolated from 2 × 10^6^ cells as described [16]. NF-κB, or p53 EMSA was performed according to The Thermo Scientific LightShift Chemiluminescent EMSA Kit (Rockford, IL) following the instructions of the manufacturer.

### Efficacy studies in nude mouse breast xenografts and orthotopic model

All animal experiments were approved by the Institutional Animal Care and Use Committee.

#### Treatment protocol

Female Balb/C nude mice (Charles River Laboratories, Wilmington, MA) were inoculated subcutaneously into each of their flanks with 2.5-3 × 10^6^ TNBC cells (MDA-MB-231 or BT-20) in Matrigel (BD Biosciences, Franklin Lakes, NJ). When the tumor reached approximately 100-150 mm^3^, animals were randomized into the control and treatment groups (n = 10/group). For MDA-MB-231 xenografts, the animals were treated with vehicle or PA-2 120 mg/kg p.o. in corn oil 5 times/wk. For BT-20 xenografts, animals were treated with vehicle or PA-2 300 mg/kg i.p. in corn oil 5 times/wk.

#### Prevention protocol

Female Balb/C nude mice were treated with PA-2 120 mg/kg or ASA 40 mg/kg p.o. in corn oil (equimolar) for 1 wk. Then, the mice were inoculated into the mammary fat pad with 1.0 × 10^6^ MDA-MB-231 cells in Matrigel. Drug treatment was continued until the end of the study. Tumor volume was calculated as [length × width × (length + width/2) × 0.56]. At the end of treatment, animals were sacrificed and tumors were removed and weighed. To calculate tumor growth inhibition, we subtracted the baseline tumor volume from the final one.

### Immunohistochemical analysis

Immunohistochemical staining for Ki-67, Dmp1 and phospho-NF-κB (p-p65, activated form of NF-κB) was performed on human breast xenograft tissue samples as previously described [18]. Apoptosis was determined by the terminal deoxynucleotidyl transferase-mediated deoxyuridine triphosphate-biotin nick end-labeling (TUNEL) assay [19].

### Statistical analysis

Results are expressed as mean ± SEM. Differences between groups were determined by one-factor analysis of variance followed by Tukey’s test for multiple comparisons. p < 0.05 was statistically significant.

## Results

### PA-2 inhibits the growth of human TNBC through a strong cytokinetic effect

We first compared the growth inhibitory effect of PA-2 and aspirin in a panel of TNBC cell lines. PA-2 inhibited cell growth more potently than aspirin in all the cell lines evaluated. The potency enhancement ranged between 5 and 7-fold in MDA-MB-231, MDA-MB-468, and BT-20 (Figure1A). PA-2 inhibited TNBC cell growth via a triple cytokinetic effect. In MDA-MB-231 and BT-20 cells, PA-2 a) inhibited cell proliferation by > 40% at 0.5 × IC_50_ and by > 80% at 1 × IC_50_; b) induced apoptosis by 1.6- to 12-fold over control at 1.5-and 2 × IC_50_; and c) suppressed the G_1_ to S cell cycle phase transition, leading to accumulation of cells in G_1_ phase by 14% at 0.5 × IC_50_ (Figure1B-D).

**Figure 2:**
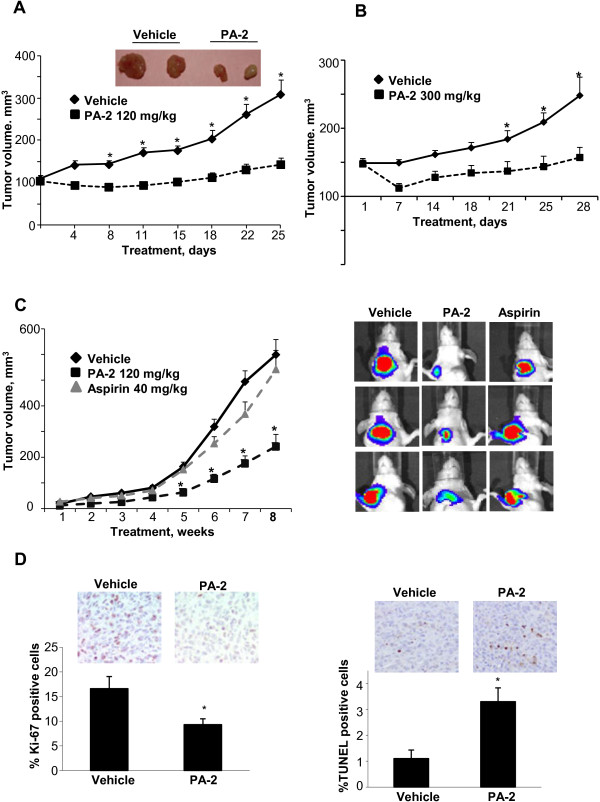
**Phospho-aspirin-2 inhibits the growth of TNBC xenografts. A:** Chemotherapeutic effect of PA-2 on subcutaneous MDA-MB-231 xenografts in nude mice. Two representative tumors from each group are shown. *, p < 0.001, compared to vehicle; *n* = 10-16 tumors/group. **B:** Chemotherapeutic effect of PA-2 on subcutaneous BT-20 xenografts in nude mice. *, p < 0.01, compared to vehicle; *n* = 10-16 tumors/group. **C:** Chemopreventive effect of PA-2. Nude mice bearing orthotopic xenografts of MDA-MB-231 cells were treated with PA-2 or aspirin for 9 wks, starting 1 wk before cell implantation. The tumor volumes of the orthotopic MDA-MB-231 xenografts at sacrifice were determined by luciferase *in vivo* imaging as described in the methods section. Representative tumors from each group are shown. *, p < 0.05, compared to vehicle. **D:** Cytokinetic effect of PA-2 in MDA-MB-231 xenografts (treatment protocol). *Left*: Representative images (top) and the quantification (bottom) of Ki-67 expression in tumor sections, * p < 0.01. *Right*: Representative images (top) and the quantification (bottom) of TUNEL positive cells in tumor sections, * p < 0.002. All values are mean ± SEM.

To assess the efficacy of PA-2 *in vivo*, we employed both subcutaneous and orthotopic TNBC xenografts in nude mice. Initially, we evaluated the *chemotherapeutic* effect of PA-2 on subcutaneous MDA-MB-231 and BT-20 xenografts. As shown in Figure 2A, PA-2 significantly inhibited MDA-MB-231 xenograft growth starting on day 8 of treatment until the end of the study (p < 0.001). At sacrifice, the tumor volume of vehicle was 309 ± 36 mm^3^ and that of PA-2 was 143 ± 16 mm^3^, representing a 79% tumor growth inhibition (p < 0.01). PA-2 also suppressed the growth of BT-20 xenografts (Figure 2B). After 28 days of treatment, the tumor volume of vehicle and PA-2 groups were 248 ± 27 mm^3^ and 157 ± 15 mm^3^, respectively (90% inhibition, p < 0.01).

We next evaluated the *chemopreventive* effect of PA-2 and compared it to aspirin, its parent compound. Following a prevention protocol, we treated nude mice bearing orthotopically implanted MDA-MB-231 xenografts with equimolar doses of PA-2 or aspirin starting 1 week before tumor implantation. On day 66 post-implantation, PA-2 inhibited the development of primary tumor in the mammary fat pads by 62% (p < 0.05; Figure 2C). In contrast, aspirin had no significant effect on breast tumor growth in this orthotopic model, consistent with previous findings [20].

We also determined cell proliferation and apoptosis in MDA-MB-231 xenografts in the treatment study (Figure 2A) using Ki-67 staining and TUNEL assay, respectively (Figure 2D). Compared to the vehicle, PA-2 inhibited cell proliferation by 44% (p < 0.01) and increased apoptosis by 3-fold (p < 0.002). This indicates that PA-2 also exerted a cytokinetic effect on TNBC xenografts *in vivo*.

### PA-2 modulates the phosphorylation status of EGFR, p53 and NF-κB

To elucidate the mechanisms of action of PA-2, we performed antibody microarray analyses (Kinexus, Vancouver, Canada) on MDA-MB-231 cells treated with vehicle or PA-2 1.5 × IC_50_ for 2 h. This assay revealed pronounced changes in EGFR, p53 and NF-κB pathways following PA-2 treatment (Additional file 1: Table S1). Therefore, we further investigate the contribution of each of these pathways to the anti-cancer effect of PA-2.

### PA-2 inhibits EGFR activation and its downstream signaling

**Figure 3:**
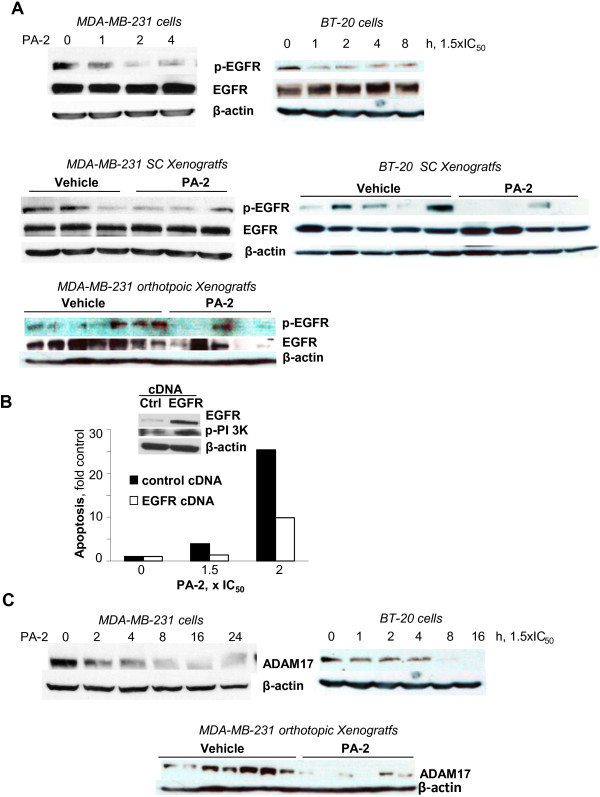
**Phospho-aspirin-2 inhibits EGFR phosphorylation. A: ***Upper:* PA-2 1.5 × IC_50_ inhibited the expression of p-EGFR in MDA-MB-231 and BT-20 cells. *Lower:* PA-2 treatment inhibited the expression of p-EFGR in MDA-MB-231 and BT-20 xenografts (p < 0.05). **B:** Effect of PA-2 at various concentrations on apoptosis in EGFR overexpressing MDA-MB-231 cells or their mock transfected control. Western blot confirmed the overexpression of EGFR and increased levels of p-PI3K. **C:** PA-2 suppressed ADAM17 levels in MDA-MB-231 and BT-20 cells in vitro and in MDA-MB-231 orthotopic xenografts (p < 0.05). In all panels, immunoblots were performed with β-actin as loading control.

EGFR is known to correlate with the progression of TNBC [11]. In MDA-MB-231 and BT-20 cells, PA-2 inhibited EGFR phosphorylation in a time-dependent manner, being evident as early as 1 h after treatment (Figure 3A). This observation was confirmed *in vivo*, where PA-2 reduced EGFR phosphorylation by 68% and 83% in MDA-MB-231 and BT-20 xenografts, respectively, compared to controls (p < 0.05, Figure 3A).

To determine the role of EGFR inhibition in the anticancer effect of PA-2, we transiently transfected MDA-MB-231 cells with an EGFR-overexpressing plasmid, and evaluated whether PA-2-induced cell death was affected. EGFR overexpression and activation of its downstream target p-PI3K was confirmed by western blot (Figure 3B). EGFR overexpression suppressed the induction of apoptosis by PA-2. Compared to mock transfected control, EGFR-overexpressing MDA-MB-231 cells have 2.5-fold reduction in the annexin V (+) fraction after treatment with PA-2 2xIC_50_ (Figure 3B). This indicates that EGFR inhibition is an important mechanism of action of PA-2, and the reversal of this effect mediates drug resistance.

An important upstream regulator of EGFR phosphorylation is ADAM17, which activates EGFR through a ligand cleavage mechanism [21]. We assessed the effect of PA-2 on ADAM proteins. As shown in Figure 2C, in MDA-MB231 and BT-20 cells, PA-2 reduced the expression of ADAM17. Moreover, PA-2 suppressed the levels of ADAM17 in MDA-MB-231 orthotopic xenografts.

**Figure 4:**
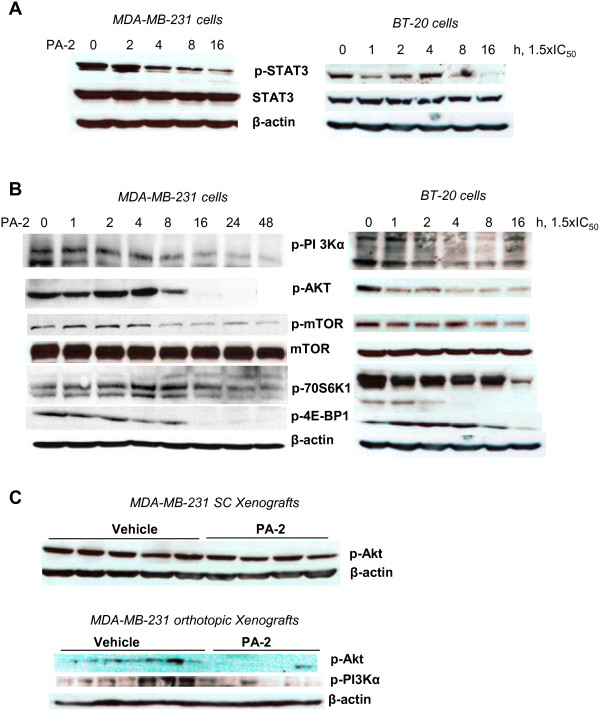
**Phospho-aspirin-2 inhibits EGFR downstream signaling. A.** PA-2 1.5 × IC_50_ inhibited STAT3 phosphorylation in MDA-MB-231 and BT-20 cells in a time-dependent manner. **B:** PA-2 treatment resulted in the sequential inactivation of PI3K signaling cascade, as indicated by the time-dependent reduction of the expression of p-PI3K, p-Akt, p-mTOR, p-p70S6K and p-4E-BP-1 in MDA-MB-231 and BT-20 cells. **C:** PA-2 reduced p-Akt expression in subcutaneous (treatment protocol) and orthotopic (prevention protocol) MDA-MB-231 xenografts. In all panels, immunoblots were performed with β-actin as loading control.

Inhibition of EGFR activation resulted in a potent inhibitory effect on its downstream signaling cascades, STAT3 and PI3K/Akt pathways. PA-2 reduced STAT3 phosphorylation in MDA-MB-231 and BT-20 cells (Figure 4A). PA-2 also suppressed the levels of p-PI3K and p-Akt in these cells *in vitro* (Figure 4B) and in MDA-MB-231 xenografts (Figure 4C). In addition, the downstream targets of PI3K/Akt pathway, including p-mTOR, p-4E-BP1 and p-70S6K1, were reduced after prolonged (16h) PA-2 treatment (Figure 4B). Hence, PA-2 triggered a temporal suppression of EGFR signaling cascades in TNBC.

### PA-2 induces acetylation of p53 and cell cycle arrest

**Figure 5:**
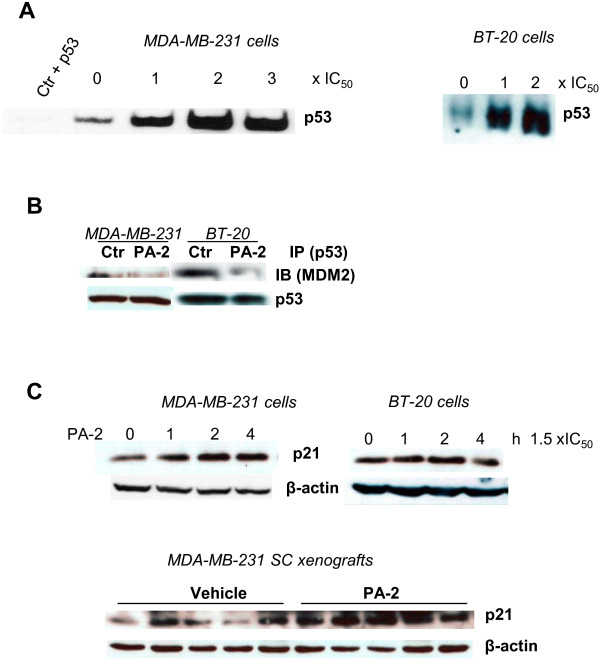
**Phospho-aspirin-2 induces p53 activity and p21. A:** PA-2 1 × IC_50_-3 × IC_50_ increased the DNA binding activity of p53 in MDA-MB-231 and BT-20 cells, as determined by electrophoretic mobility shift assay. **B:** PA-2 1.5 × IC_50_ disrupted the interaction between p53 and MDM2 in MDA-MB-231 and BT-20 cells. Following treatment with PA-2, p53 was immunoprecipitated and the levels of MDM2 were determined. **C: ***Upper:* PA-2 1.5 × IC_50_ induced the expression of p21 in MDA-MB-231 and BT-20 cells. *Lower*: PA-2 induced the expression of p21 in MDA-MB-231 xenografts (treatment protocol).

The tumor suppressor gene p53 is frequently inactivated in TNBC [22]. In MDA-MB-231 and BT-20 cells, PA-2 enhanced the DNA-binding activity of p53 in a concentration-dependent manner (Figure 5A). PA-2 did not appear to alter the nuclear shuttling of p53 (Figure 5A). On the other hand, immunoprecipitation showed that PA-2 significantly reduced the binding of p53 to murine double minute 2 (MDM2) (Figure 5B). Dissociation of p53 from MDM2, which otherwise binds to p53 and represses its transcriptional activity [23], may therefore contribute to the induction of p53 DNA binding activity by PA-2 in TNBC cells. The activation of p53 by PA-2 was consequential, as PA-2 blocked G_1_ to S cell cycle transition (Figure 1D) and up-regulated p21 in TNBC cells *in vitro* and in MDA-MB-231 xenografts (Figure 5C).

**Figure 6:**
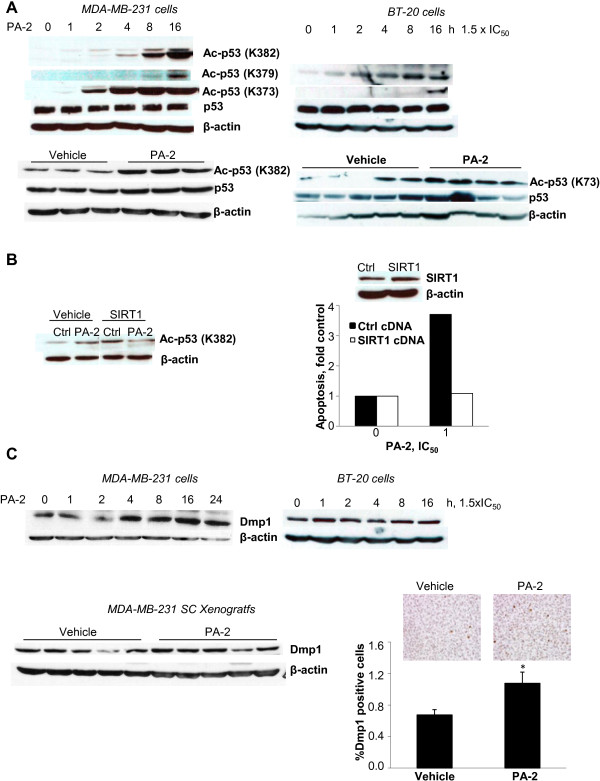
**Phospho-aspirin-2 induces p53 acetylation and Dmp1 expression. A: ***Upper:* PA-2 induced the acetylation of p53 in MDA-MB-231 (K373, K379 and K382) and BT-20 (K373 and K379) cells. *Lower:* PA-2 induced p53 acetylation in MDA-MB-231 (K382) and BT-20 (K373) xenografts. **B: ***Left panel:* SIRT1 overexpression prevents p53 acetylation (K382) by PA-2. *Right panel:* SIRT1 overexpression attenuated PA-2-induced apoptosis in MDA-MB-231 cells. Western blot confirmed the overexpression of SIRT1. **C: ***Upper:* PA-2 1.5 x IC_50_ increased the expression of Dmp1 in MDA-MB-231 and BT-20 cells. *Lower*: PA-2 increased Dmp1 expression in xenografts, as indicated by western blot (*left*) and immunohistochemistry (*right*). Two representative tissue sections are shown. *, p < 0.02, compared to control; magnification 200X. Immunoblots were performed with β-actin as the loading control.

Acetylation of p53 at lysine residues is critical for its stability and transcriptional activity [24]. Given that the aspirin moiety of PA-2 contains an acetyl group capable of acetylating multiple proteins in cancer cells [25], we examined the effect of PA-2 on the acetylation status of p53. In MDA-MB-231 cells, PA-2 induced p53 acetylation at three distinct lysine residues (K373, K379 and K382) in a time-dependent manner; while in BT-20 cells PA-2 induced acetylation at K373 and K379 residues (Figure 6A). In MDA-MB-231 and BT-20 xenografts, treatment with PA-2 increased p53 acetylation at K382 and K373 residues, respectively (Figure 6A).

To further assess the role of p53 acetylation in cell death induction by PA-2, we overexpressed in MDA-MB-231 cells SIRT1, which negatively regulates p53 through its de-acetylation [26,27]. Overexpression of SIRT1 blocked the ability of PA-2 to acetylate p53 at the K382 residue (Figure 6B). Importantly, SIRT1 overexpression attenuated the induction of apoptosis in response to PA-2 by 71%, indicating that PA-2 induces apoptosis, at least in part, by a p53 acetylation-dependent mechanism (Figure 6B).

PA-2 may also regulate p53 independently of acetylation. PA-2 significantly enhanced the expression of Dmp1, a tumor suppressor that induces p53-dependent cell cycle arrest by directly binding to its promoter [28]. Such an effect is observed in TNBC cells *in vitro.* In MDA-MB-231 xenografts, PA-2 treatment increased Dmp1 expression by 57% (p < 0.02) compared to the control group (Figure 6C).

### PA-2 induces RONS levels, inhibits the thioredoxin system and NF-κB activation

**Figure 7:**
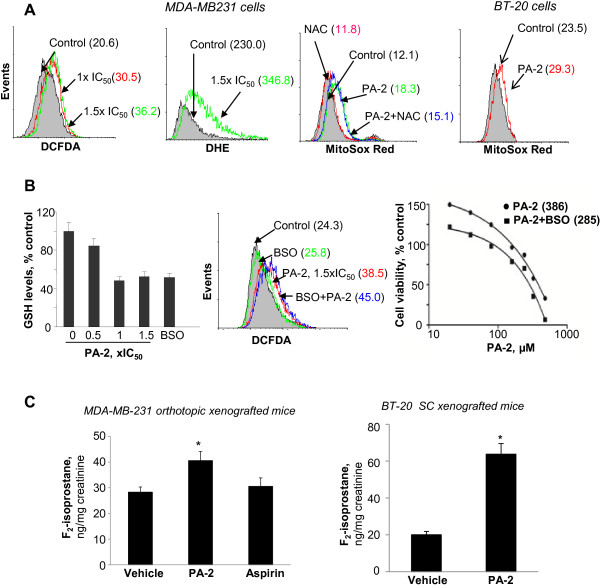
**Phospho-aspirin-2 induces oxidative stress in TNBC. A:** PA-2 induced RONS in MDA-MB-231 and BT-20 cells after 1h treatment, as determined by DCFDA, DHE and MitoSOX Red staining and flow cytometry. **B: ***Left panel:* GSH level was suppressed in MDA-MB-231 cells treated with various concentrations of PA-2 for 24 h, BSO as a positive control. Values are mean ± SEM. *Middle panel:* PA-2 and BSO synergistically induced RONS. RONS production was determined by DCFDA staining in MDA-MB-231 cells treated with PA-2 or PA-2 plus BSO for 1 h. *Right panel:* PA-2 and BSO synergistically inhibited cell growth. Cell growth inhibition was determined by MTT in MDA-MB-231 cells treated with PA-2 or PA-2 plus BSO for 24h. **C:** PA-2 increased the levels of 15-F_2t_-Isoprostane in 24-h urine from nude mice bearing orthotopic MDA-MB231 (p < 0.05) and subcutaneous BT-20 xenografts (p < 0.006), while aspirin had no effect. Urinary 15-F_2t_-Isoprostane was determined using an ELISA kit, as described in Methods.

RONS play a significant role in the action of phospho-NSAIDs [29]. We determined the effect of PA-2 using various molecular probes: DCFDA (general RONS), DHE (cytoplasmic O_2_•^−^), and MitoSOX Red (mitochondrial O_2_•^−^). Compared to control, PA-2 1.5 × IC_50_ increased DCFDA by 76%, DHE by 51% and MitoSOX Red by 51% in MDA-MB-231 cells (Figure 7A). N-acetylcysteine (10 mM), a ROS scavenger, partly blocked ROS induction by 26% in MDA-MB-231 cells (Figure 7A). In BT-20 cells, PA-2 increased MitoSOX Red by 25%. PA-2 1-1.5 × IC_50_ also decreased the level of glutathione, a major cellular antioxidant. Co-incubation of PA-2 with BSO, an inhibitor of GSH synthesis, synergistically induced RONS levels and inhibited cell growth (Figure 7B).

To assess the effect of PA-2 on RONS *in vivo*, we measured urinary 15-F_2t_-isoprostane, a marker of oxidative stress [30,31], in the mice bearing TNBC xenografts. In orthotopic MDA-MB-231 xenografts (Figure 2C), 15-F_2t_-isoprostane levels on day 25 were 28.7 ± 3.3 ng/mg creatinine in controls and 40.9 ± 2.2 ng/mg creatinine in the PA-2 group, representing a nearly 40% increase (p < 0.05) (Figure 7C). In contrast, aspirin had no significant effect (p = 0.6). In BT-20 xenografts, PA-2 treatment increased 15-F_2t_-isoprostane levels by over 3-fold over the control (p < 0.006). Hence, PA-2, but not aspirin, induced RONS *in vivo*.

**Figure 8:**
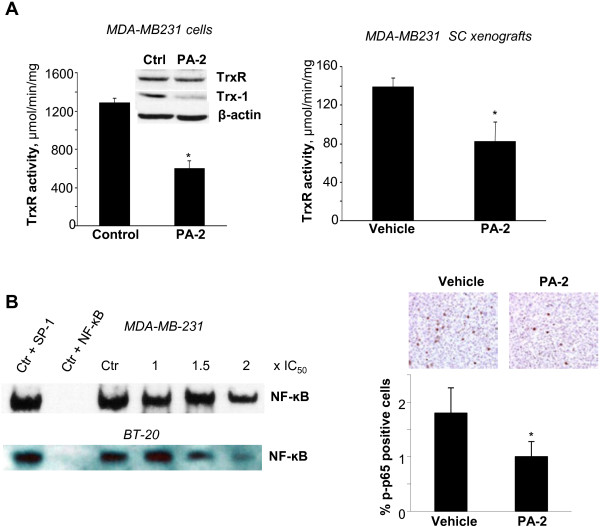
**Phospho-aspirin-2 suppresses the thioredoxin (Trx) system and activation of NF-кB. A: ***Left panel:* PA-2 1.5 × IC_50_ reduced TrxR activity in MDA-MB-231 cells after 1 h treatment. *, p < 0.02, compared to control. Immunoblots of TrxR and Trx-1 showed that PA-2 reduced the expression of Trx-1 in MDA-MB-231 cells. *Right panel:* PA-2 reduced TrxR activity in the protein lysates from MDA-MB-231 xenografts (treatment protocol) from animals treated with vehicle or PA-2 for 25 days. *, p < 0.04, compared to vehicle. **B: ***Left panel:* PA-2 inhibited constitutive NF-κB activation. EMSA for NF-κB of nuclear fractions isolated from MDA-MB-231 (*upper*) and BT-20 (*lower*) cells after 4 h treatment with or without PA-2 1.5 × IC_50_. To determine the specificity of the NF-κB transcription factor-DNA complex, the control nuclear fraction was incubated in the presence of 100-fold molar excess of unlabeled oligonucleotide containing the consensus sequence for either the specific (+NF-κB) or an unspecific (+AP-1) transcription factor. *Right panel:* NF-κB (p-p65) levels from MDA-MB-231 tumors, determined by immunohistochemistry using an anti-p-p65 antibody, were reduced in PA-2 treated group compared to the vehicle control. The percentage of p-p65-positive cells in various fields was determined and averaged for each xenograft. *, p < 0.0009, compared to vehicle. Representative images are shown; magnification 200X. All values are mean ± SEM.

The thioredoxin (Trx) system, composed of thioredoxin reductase (TrxR) and Trx-1, plays an important role in redox homeostasis by reducing oxidized proteins; the latter is overexpressed in TNBC [32]. PA-2 inhibited TrxR activity in MDA-MB-231 cells in cell culture and MDA-MB-231 xenografts by 54% and 41%, respectively (p < 0.02-0.04), without affecting its levels (Figure 8A). PA-2 also significantly reduced Trx-1 levels in MDA-MB-231 cells after 1h treatment with 1.5 × IC_50_ PA-2 (Figure 8A). These results indicate that PA-2 targets major components of the Trx system.

The Trx system is closely linked with the NF-κB signaling pathway. Trx-1 enhances DNA binding of NF-κB by reducing the intermolecular Cys62 -S-S- bond of its p50 subunit [33]. We thus examined the effect on PA-2 on NF-κB activation. Consistent with its inhibitory effect on Trx-1 expression, PA-2 also inhibited NF-κB-DNA binding in a concentration-dependent manner in MDA-MB-231 and BT-20 cells (Figure 8B). A similar inhibition was observed in MDA-MB-231 xenografts. PA-2 reduced the levels of activated p-p65 by 44% (p < 0.009), compared to the control group (Figure 8B).

## Discussion

Our data demonstrate that PA-2, a novel derivative of aspirin, effectively inhibits TNBC in preclinical models and is much more potent than aspirin, its parent compound. The anticancer activity of PA-2 is associated with a pronounced effect on a) EGFR activation; b) p53 acetylation and c) RONS induction. These signaling effects of PA-2 culminate in the substantial inhibition of cell proliferation and induction of apoptosis, the net effect of which is a strong reduction in TNBC xenograft growth *in vivo*.

Efficacy and safety are the prime considerations in the evaluation of anticancer agents [34], and the latter is especially paramount in chemoprevention. Extensive epidemiological and clinical evidence supports a beneficial role of aspirin in the prevention of breast, colon, and lung cancers [35]; however, its gastrointestinal toxicity is limiting. Our results demonstrated that PA-2, with the novel phospho-modification, is superior to aspirin both in terms of efficacy in TNBC, as well as safety [9]. PA-2, but not aspirin, administered in a chemoprevention protocol slowed the development of orthotopic MDA-MB-231 xenografts.

PA-2 is highly efficacious when used in the treatment setting and its chemotherapeutic effect is even stronger than its chemopreventive effect. PA-2 potently inhibits TNBC *in vivo*, almost completely arresting the growth of both MDA-MB-231 and BT-20 xenografts. Consistent with its strong chemotherapeutic efficacy, PA-2 induced a profound cytokinetic effect involving inhibition of cell proliferation and induction of apoptosis. Hence, PA-2 is a promising anticancer candidate that merits further evaluation.

**Figure 9:**
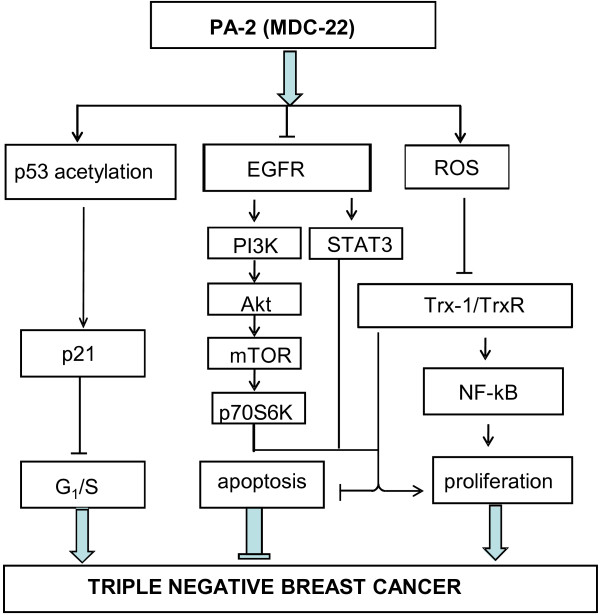
**Proposed mechanism for the anticancer effect of phospho-aspirin-2 in TNBC.** PA-2 inhibits TNBC through (i) inhibition of EGFR phosphorylation and attenuation of downstream signaling cascades (STAT3 and PI3K/Akt); (ii) acetylation of p53, which enhances p53 DNA-binding activity, p21 expression and cell cycle arrest; and (iii) induction of oxidative stress and alteration of the Trx system; which culminate in the inhibition of cell proliferation and induction of apoptosis in TNBC.

Our work identified EGFR, p53 and RONS as the major signaling mechanisms (Figure 9) involved in eliciting the growth inhibitory effect of PA-2. EGFR is overexpressed in 16–48% of breast cancers and its expression is associated with poor prognosis [36,37]. In particular, EGFR is frequently overexpressed in TNBC, a subset of breast cancer that is characterized by their unique molecular profile, aggressive behavior and distinct patterns of metastasis [38]. PA-2 is an inhibitor of EGFR phosphorylation *in vitro* and *in vivo*. The inhibitory effect of PA-2 appears to be mediated by a novel mechanism involving the inhibition of ADAM17, a major protease that controls availability of EGFR ligands [39]. A key consequence of PA-2-induced inhibition of EGFR is the suppression of pro-survival STAT3 and PI3K and the sequential inactivation of their downstream signaling propagation, thereby causing growth inhibition and cell death.

Mutations of p53 are exceptionally frequent in TNBC (>80%) and its inactivation predicts poor survival in TNBC patients [10,12]. Activation-inactivation of p53 depends on a repertoire of post-translational modifications [40], including phosphorylation and acetylation. p53 acetylation was found to be indispensable for its activation, as it destabilizes the p53-MDM2 interaction, thereby abrogating MDM2-mediated transcription repression [24]. Here, we show that PA-2 acetylates p53 *in vitro* and *in vivo* and disrupts its association with MDM2, which in turn, enhances p53-DNA binding activity. Forced deacetylation of p53 partly attenuated cell death induction by PA-2, indicating an important role of p53 acetylation in mediating the effect of PA-2. PA-2 additionally regulates p53 independently of acetylation. Dmp1 is a transcription factor that physically interacts with p53 [41]. Dmp1-p53 binding antagonizes the ubiquitination of p53 byMDM-2 and promotes the nuclear translocation of p53 [28]. PA-2 enhanced the expression of Dmp1 in TNBC cells and in xenografts, which may further contribute to the robust p53 activation triggered by PA-2.

The induction of oxidative stress plays a key role in the anticancer effect of structurally related phospho-NSAIDs [9,42]. PA-2 induces oxidative stress in TNBC cells in culture and in xenografts by compromising cellular antioxidant defense mechanisms. First, PA-2 suppressed the Trx system, with both TrxR and Trx-1 affected in a significant way. TrxR and Trx-1 are critical components of the cellular redox system [43]. Trx-1 reduces client proteins oxidized by RONS, with itself undergoing oxidation, while TrxR regenerates the reduced Trx-1. In TNBC cells and xenografts, PA-2 inhibited TrxR activity and decreased the expression of Trx-1. Second, PA-2 significantly reduced intracellular levels of GSH, the major chemical antioxidant of mammalian cells. Induction of oxidative stress has major repercussions on redox- and Trx-dependent signaling cascades, as exemplified by the inhibitory effect on NF-кB. NF-кB is constitutively active in TNBC [44], and its aberrant activation is linked to inflammation and cancer. Induction of oxidative stress and the subsequent inhibition of NF-кB by PA-2 may mediate part of its growth inhibitory effect.

It is of interest that PA-2 has superior efficacy compared to conventional aspirin, the starting compound for its synthesis. The reasons for this difference are not entirely clear, but as a new chemical entity, PA-2 is expected to have distinct properties from its parent compound. Perhaps the most relevant property of these two compounds is their ability to induce oxidative stress, an effect that accounts for much of the anticancer efficacy of phospho-NSAIDs, including phospho-aspirin [29]. Indeed, PA-2 caused robust oxidative stress in mice, reflected in enhanced urinary levels of the biomarker F_2_-isoprostane. In contrast, aspirin failed to produce such an effect, and this difference explains to a large extent their differential efficacy. Additional mechanistic differences might also contribute, especially with regards to effects on cell signaling cascades.

## Conclusions

Our work indicates that PA-2 possesses chemotherapeutic and chemopreventive efficacy against TNBC in preclinical models, and establishes inhibition of EGFR, acetylation of p53 and induction of oxidative stress as critical mediators of its mechanism of action (Figure 9). The multi-targeted nature of PA-2 towards the dysregulated signaling cascades in TNBC further suggests the notion that PA-2 may be a promising therapeutic option, either alone or in combination with other therapies.

## Abbreviations

ADAM: A disintegrin and metalloproteinase; Akt: Protein kinase B; EGFR: Epidermal growth factor receptor; ER: Estrogen receptor; mTOR: Mammalian target of rapamycin; NSAID: Nonsteroidal anti-inflammatory drug; NF-ΚB: Nuclear factor kappa-light-chain-enhancer of activated B cells; PA-2: Phospho-aspirin-2; PI3K: Phosphoinositide 3-kinase; PR: Progesterone receptor; RONS: Reactive oxygen and nitrogen species; SIRT1: Sirtuin; STAT3: Signal transducer and activator of transcription 3; Trx-1: Thioredoxin-1; TrxR: Thioredoxin reductase; SC: Subcutaneous.

## Competing interests

The authors have nothing to disclose except for BR, who has an equity position in Medicon Pharmaceuticals, Inc. and NO who is an employee for the same.

## Authors’ contributions

LH conceived the study, participated in its design, carried out most of the in vitro and in vivo studies, analyzed the data and participated in the preparation of the manuscript. YS participated in the study design, performed the orthotopic transplantation of breast cancer cells and involved in the drafting the manucript; CCW, GGM, KWC participated in the study design, data analyses and writing of the manuscript. KV synthesized batches of PA-2, participated in the study design and data analysis. NA performed the luciferase imaging of breast tumors, participated in data collection and data analysis. NO performed the immunohistochemical analyses, participated in data collection and data analysis. BR participated in the study design, supervised the work, analyzed data and participated in writing the manuscript. All authors read and approved the final manuscript.

## Pre-publication history

The pre-publication history for this paper can be accessed here:

http://www.biomedcentral.com/1471-2407/14/141/prepub

## Supplementary Material

Additional file 1: Table S1 Antibody microarray analysis on phosphor-aspirin treated MDA-MB-231 cells. TNBC MDA-MB-231 cells treated with vehicle or PA-2 1.5 × IC_50_ for 2 h were performed antibody microarray analysis by *Kinexus* (Vancouver, CA)*.* Click here for file

## References

[B1] Siegel R Naishadham D Jemal A Cancer statistics, 2012 CA Cancer J Clin 2012 62 1 10 29 10.3322/caac.20138 22237781

[B2] Rinsema TJ One hundred years of aspirin Med Hist 1999 43 4 502 507 10.1017/S0025727300065728 10885147 PMC1044183

[B3] Stanley P Hegedus R Aspirin–the first hundred years Biologist (London) 2000 47 5 269 271 11153137

[B4] Baron JA What now for aspirin and cancer prevention? J Natl Cancer Inst 2004 96 1 4 5 10.1093/jnci/djh027 14709726

[B5] Baron JA Cole BF Sandler RS Haile RW Ahnen D Bresalier R McKeown-Eyssen G Summers RW Rothstein R Burke CA Snover DC Church TR Allen JI Beach M Beck GJ Bond JH Byers T Greenberg ER Mandel JS Marcon N Mott LA Pearson L Saibil F van Stolk RU A randomized trial of aspirin to prevent colorectal adenomas N Engl J Med 2003 348 10 891 899 10.1056/NEJMoa021735 12621133

[B6] Marshall SF Bernstein L Anton-Culver H Deapen D Horn-Ross PL Mohrenweiser H Peel D Pinder R Purdie DM Reynolds P Stram D West D Wright WE Ziogas A Ross RK Nonsteroidal anti-inflammatory drug use and breast cancer risk by stage and hormone receptor status J Natl Cancer Inst 2005 97 11 805 812 10.1093/jnci/dji140 15928301

[B7] Piazza GA Keeton AB Tinsley HN Gary BD Whitt JD Mathew B Thaiparambil J Coward L Gorman G Li Y Sani B Hobrath JV Maxuitenko YY Reynolds RC A novel sulindac derivative that does not inhibit cyclooxygenases but potently inhibits colon tumor cell growth and induces apoptosis with antitumor activity Cancer Prev Res (Phila) 2009 2 6 572 580 10.1158/1940-6207.CAPR-09-0001 19470791 PMC3227417

[B8] Wong CC Cheng KW Xie G Zhou D Zhu CH Constantinides PP Rigas B Carboxylesterases 1 and 2 hydrolyze phospho-nonsteroidal anti-inflammatory drugs: relevance to their pharmacological activity J Pharmacol Exp Ther 2012 340 2 422 432 10.1124/jpet.111.188508 22085648 PMC3263964

[B9] Huang L Mackenzie G Ouyang N Sun Y Xie G Johnson F Komninou D Rigas B The novel phospho-non-steroidal anti-inflammatory drugs, OXT-328, MDC-22 and MDC-917, inhibit adjuvant-induced arthritis in rats Br J Pharmacol 2011 162 7 1521 1533 10.1111/j.1476-5381.2010.01162.x 21175575 PMC3057290

[B10] Gasco M Shami S Crook T The p53 pathway in breast cancer Breast Cancer Res 2002 4 2 70 76 10.1186/bcr426 11879567 PMC138723

[B11] Price JT Tiganis T Agarwal A Djakiew D Thompson EW Epidermal growth factor promotes MDA-MB-231 breast cancer cell migration through a phosphatidylinositol 3′-kinase and phospholipase C-dependent mechanism Cancer Res 1999 59 21 5475 5478 10554021

[B12] Cancer Genome Atlas Network Comprehensive molecular portraits of human breast tumours Nature 2012 490 7418 61 70 10.1038/nature11412 23000897 PMC3465532

[B13] Dancey JE Freidlin B Targeting epidermal growth factor receptor–are we missing the mark? Lancet 2003 362 9377 62 64 10.1016/S0140-6736(03)13810-X 12853203

[B14] Vousden KH Prives C Blinded by the light: the growing complexity of p53 Cell 2009 137 3 413 431 10.1016/j.cell.2009.04.037 19410540

[B15] Zhao W Mackenzie GG Murray OT Zhang Z Rigas B Phosphoaspirin (MDC-43), a novel benzyl ester of aspirin, inhibits the growth of human cancer cell lines more potently than aspirin: a redox-dependent effect Carcinogenesis 2009 30 3 512 519 10.1093/carcin/bgp015 19136474 PMC2650796

[B16] Mackenzie GG Sun Y Huang L Xie G Ouyang N Gupta RC Johnson F Komninou D Kopelovich L Rigas B Phospho-sulindac (OXT-328), a novel sulindac derivative, is safe and effective in colon cancer prevention in mice Gastroenterology 2010 139 4 1320 1332 10.1053/j.gastro.2010.06.044 20600034 PMC2949489

[B17] Hundley TR Gilfillan AM Tkaczyk C Andrade MV Metcalfe DD Beaven MA Kit and FcepsilonRI mediate unique and convergent signals for release of inflammatory mediators from human mast cells Blood 2004 104 8 2410 2417 10.1182/blood-2004-02-0631 15217825

[B18] Ouyang N Williams JL Rigas B NO-donating aspirin isomers downregulate peroxisome proliferator-activated receptor (PPAR){delta} expression in APCmin/+ mice proportionally to their tumor inhibitory effect: Implications for the role of PPAR{delta} in carcinogenesis Carcinogenesis 2006 27 2 232 239 10.1093/carcin/bgi221 16141240

[B19] Rigas B Kozoni V The novel phenylester anticancer compounds: Study of a derivative of aspirin (phoshoaspirin) Int J Oncol 2008 32 1 97 100 18097547

[B20] Likhite V Aspirin and Breast Cancer: Studies In Mice Central Reginoal Meeting of the American Chemical Society 2009 Cleveland, Ohio

[B21] Baumgart A Seidl S Vlachou P Michel L Mitova N Schatz N Specht K Koch I Schuster T Grundler R Kremer M Fend F Siveke JT Peschel C Duyster J Dechow T ADAM17 regulates epidermal growth factor receptor expression through the activation of Notch1 in non-small cell lung cancer Cancer Res 2010 70 13 5368 5378 10.1158/0008-5472.CAN-09-3763 20551051

[B22] Soussi T Lozano G p53 mutation heterogeneity in cancer Biochem Biophys Res Commun 2005 331 3 834 842 10.1016/j.bbrc.2005.03.190 15865939

[B23] Meek DW Anderson CW Posttranslational modification of p53: cooperative integrators of function Cold Spring Harb Perspect Biol 2009 1 6 a000950 20457558 10.1101/cshperspect.a000950 PMC2882125

[B24] Tang Y Zhao W Chen Y Zhao Y Gu W Acetylation is indispensable for p53 activation Cell 2008 133 4 612 626 10.1016/j.cell.2008.03.025 18485870 PMC2914560

[B25] Marimuthu S Chivukula RS Alfonso LF Moridani M Hagen FK Bhat GJ Aspirin acetylates multiple cellular proteins in HCT-116 colon cancer cells: identification of novel targets Int J Oncol 2011 39 5 1273 1283 21743961 10.3892/ijo.2011.1113

[B26] Ito A Kawaguchi Y Lai CH Kovacs JJ Higashimoto Y Appella E Yao TP MDM2-HDAC1-mediated deacetylation of p53 is required for its degradation EMBO J 2002 21 22 6236 6245 10.1093/emboj/cdf616 12426395 PMC137207

[B27] Solomon JM Pasupuleti R Xu L McDonagh T Curtis R DiStefano PS Huber LJ Inhibition of SIRT1 catalytic activity increases p53 acetylation but does not alter cell survival following DNA damage Mol Cell Biol 2006 26 1 28 38 10.1128/MCB.26.1.28-38.2006 16354677 PMC1317617

[B28] Taneja P Maglic D Kai F Sugiyama T Kendig RD Frazier DP Willingham MC Inoue K Critical roles of DMP1 in human epidermal growth factor receptor 2/neu-Arf-p53 signaling and breast cancer development Cancer Res 2010 70 22 9084 9094 10.1158/0008-5472.CAN-10-0159 21062982 PMC3073839

[B29] Sun Y Huang L Mackenzie GG Rigas B Oxidative stress mediates through apoptosis the anticancer effect of phospho-nonsteroidal anti-inflammatory drugs: implications for the role of oxidative stress in the action of anticancer agents J Pharmacol Exp Ther 2011 338 3 775 783 10.1124/jpet.111.183533 21646387 PMC3164348

[B30] Basu S F2-isoprostanes in human health and diseases: from molecular mechanisms to clinical implications Antioxid Redox Signal 2008 10 8 1405 1434 10.1089/ars.2007.1956 18522490

[B31] Tacconelli S Capone ML Patrignani P Measurement of 8-iso-prostaglandin F2alpha in biological fluids as a measure of lipid peroxidation Methods Mol Biol 2010 644 165 178 10.1007/978-1-59745-364-6_14 20645173

[B32] Mukherjee A Martin SG The thioredoxin system: a key target in tumour and endothelial cells Br J Radiol 2008 81 Spec No 1 S57 S68 18819999 10.1259/bjr/34180435

[B33] Matthews JR Wakasugi N Virelizier JL Yodoi J Hay RT Thioredoxin regulates the DNA binding activity of NF-kappa B by reduction of a disulphide bond involving cysteine 62 Nucleic Acids Res 1992 20 15 3821 3830 10.1093/nar/20.15.3821 1508666 PMC334054

[B34] Wong CC Cheng KW Rigas B Preclinical predictors of anticancer drug efficacy: critical assessment with emphasis on whether nanomolar potency should be required of candidate agents J Pharmacol Exp Ther 2012 341 3 572 578 10.1124/jpet.112.191957 22448039 PMC3362883

[B35] Cuzick J Otto F Baron JA Brown PH Burn J Greenwald P Jankowski J La Vecchia C Meyskens F Senn HJ Thun M Aspirin and non-steroidal anti-inflammatory drugs for cancer prevention: an international consensus statement Lancet Oncol 2009 10 5 501 507 10.1016/S1470-2045(09)70035-X 19410194

[B36] Tsutsui S Kataoka A Ohno S Murakami S Kinoshita J Hachitanda Y Prognostic and predictive value of epidermal growth factor receptor in recurrent breast cancer Clin Cancer Res 2002 8 11 3454 3460 12429634

[B37] Tsutsui S Ohno S Murakami S Hachitanda Y Oda S Prognostic value of epidermal growth factor receptor (EGFR) and its relationship to the estrogen receptor status in 1029 patients with breast cancer Breast Cancer Res Treat 2002 71 1 67 75 10.1023/A:1013397232011 11859875

[B38] Nielsen TO Hsu FD Jensen K Cheang M Karaca G Hu Z Hernandez-Boussard T Livasy C Cowan D Dressler L Akslen LA Ragaz J Gown AM Gilks CB van de Rijn M Perou CM Immunohistochemical and clinical characterization of the basal-like subtype of invasive breast carcinoma Clin Cancer Res 2004 10 16 5367 5374 10.1158/1078-0432.CCR-04-0220 15328174

[B39] Sternlicht MD Sunnarborg SW The ADAM17-amphiregulin-EGFR axis in mammary development and cancer J Mammary Gland Biol Neoplasia 2008 13 2 181 194 10.1007/s10911-008-9084-6 18470483 PMC2723838

[B40] Walerych D Napoli M Collavin L Del Sal G The rebel angel: mutant p53 as the driving oncogene in breast cancer Carcinogenesis 2012 33 11 2007 2017 10.1093/carcin/bgs232 22822097 PMC3483014

[B41] Frazier DP Kendig RD Kai F Maglic D Sugiyama T Morgan RL Fry EA Lagedrost SJ Sui G Inoue K Dmp1 physically interacts with p53 and positively regulates p53′s stability, nuclear localization, and function Cancer Res 2012 72 7 1740 1750 10.1158/0008-5472.CAN-11-2410 22331460 PMC3319807

[B42] Sun Y Rigas B The thioredoxin system mediates redox-induced cell death in human colon cancer cells: implications for the mechanism of action of anticancer agents Cancer Res 2008 68 20 8269 8277 10.1158/0008-5472.CAN-08-2010 18922898 PMC3565581

[B43] Kim SJ Miyoshi Y Taguchi T Tamaki Y Nakamura H Yodoi J Kato K Noguchi S High thioredoxin expression is associated with resistance to docetaxel in primary breast cancer Clin Cancer Res 2005 11 23 8425 8430 10.1158/1078-0432.CCR-05-0449 16322305

[B44] Yamamoto M Taguchi Y Ito-Kureha T Semba K Yamaguchi N Inoue J NF-kappaB non-cell-autonomously regulates cancer stem cell populations in the basal-like breast cancer subtype Nat Commun 2013 4 2299 23934482 10.1038/ncomms3299

